# Decoding Biological Age: Emerging Insights, Persistent Challenges, and Our Journey Toward De-Development or Reliable Aging Biomarkers

**DOI:** 10.1093/geroni/igaf122.1670

**Published:** 2025-12-31

**Authors:** Andrea Cipriano

**Affiliations:** Stanford University, Palo Alto, California, United States

## Abstract

Biological age provides a more accurate measure of healthspan and disease risk than chronological age, yet the development of robust and clinically actionable biomarkers remains an ongoing challenge. In this presentation, I will outline emerging insights that are reshaping our understanding of the aging process, while highlighting the persistent technical and conceptual barriers that hinder biomarker validation and standardization. Drawing on work from the Biomarkers of Aging Consortium, I will share our collaborative efforts to define, benchmark, and harmonize key readouts of biological age, with a particular focus on clinical immune aging biomarkers as a case study for translating discovery into real-world applications.

